# Salvage of severe knee osteoarthritis: efficacy of tibial condylar valgus osteotomy versus open wedge high tibial osteotomy

**DOI:** 10.1186/s13018-021-02597-x

**Published:** 2021-07-14

**Authors:** Xiaoyu Wang, Li Shi, Rui Zhang, Wenbo Wang, Lingchi Kong, Haoyu Zhao, Jia Xu, Qinglin Kang

**Affiliations:** Department of Orthopaedic Surgery, Shanghai Jiao Tong University Affiliated Sixth People’s Hospital, 600 Yishan Road, Shanghai, 200233 China

**Keywords:** Osteoarthritis (OA), Open wedge high tibial osteotomy (OWHTO), Tibial condylar valgus osteotomy (TCVO)

## Abstract

**Introduction:**

To compare the clinical outcomes and the radiographic features between tibial condylar valgus osteotomy (TCVO) and open wedge high tibial osteotomy (OWHTO). New insight into the indication criteria for TCVO was also clarified for achieving satisfactory results.

**Materials and methods:**

Sixty-three knees with medial-compartment osteoarthritis were retrospectively studied. Thirty-four knees with subluxated lateral joint and depression of the medial tibial plateau underwent TCVO and the rest underwent OWHTO. Among the 63 knees included, 27 knees with a pre-operative femorotibial angle (FTA) ≥ 185° were defined as severe varus (subgroup S, 15 in S_TCVO_ group and 12 in S_HTO_ group). Lower limb alignment, intra-, and extra-articular congruency were evaluated according to the radiograph obtained before and 24 months after surgery. The visual analog scale (VAS) score and Hospital for Special Surgery (HSS) score were obtained to assess the clinical results. Opening angle and distance of the opening gap in each group were measured by intra-operative fluoroscopy.

**Results:**

During the 2-year follow-up period, the mean HSS score increased from 70.3 to 81.4 in HTO group and 65.9 to 87.3 in TCVO group (*p* < 0.05). The mean VAS score decreased from 5.9 to 2.6 and 6.0 to 2.1, respectively (*p* < 0.01). Pre-operative FTA was restored to 172.9° in HTO group and 171.3° in TCVO group, and percentage of mechanical axis (%MA) was improved to 59.7% and 61.2%, respectively. Joint line convergence angle (JLCA) was slightly restored and medial tibial plateau depression (MTPD) was relatively the same before and after OWHTO, while these parameters improved greatly (from 6.4° to 1.2° and − 8.0° to 5.9°, *p* < 0.01) in TCVO group. More undercorrected knees were observed in S_HTO_ group than S_TCVO_ group (58.3% and 13.3%, *p* < 0.05). Opening angle and distance of the opening gap were larger in TCVO group (19.1° and 14.0 mm) than those in OWHTO group (9.3° and 10.1 mm, *p* < 0.05).

**Conclusion:**

Compared to OWHTO, TCVO had priority in treating advanced knee OA with intra-articular deformity. However, TCVO had a limited capacity to correct the varus angle. Besides, TCVO might be suitable for medial-compartment OA with a pre-operative FTA ≥ 185°.

## Introduction

Osteoarthritis (OA) of the knee is a common joint disorder which may induce lower limb deformity and joint degeneration in serious conditions, resulting in a major drop in quality of life [1]. For the patients with knee OA refractory to conservative treatment, surgical treatment is highly recommended. Although total and unicompartmental knee arthroplasty (TKA and UKA) are widely accepted as suitable procedures for severe OA, prosthesis-associated infections and wear of the implant still remain hindrances to the final outcomes. Moreover, post-operative joint range of motion (ROM) in young or physically active patients is usually beyond satisfaction [2].

Renewed knowledge in the relationship between malalignment and the development of knee OA led to the focus on osteotomies. Lateral closed-wedge high tibial osteotomy is an established technique in correcting varus deformity of the proximal tibia, which could avoid or postpone knee arthroplasty. However, this technique is considered a demanding one due to significant complications such as risk of neurovascular injuries, tibiofibular joint disruption, and compartment syndrome [3, 4]. With the development of specially designed locking-compression-fixation technique and availability of superior initial stability, medial open-wedge high tibial osteotomy (OWHTO) has gradually replaced close-wedge osteotomy procedure in treating varus deformed knees, especially for physically active patients who desire knee joint preservation [3, 5, 6].

Extra- and intra-articular pathologies are two major types of OA. Extra-articular varus knee arthrosis is usually derived from bony causes, leading to relatively mild lesion to the soft tissue of the knee joint [7]. Intra-articular varus deformity usually occurs in severe OA with aggravated osseocartilaginous wearing, which causes joint instability and abnormal joint kinematics during exercise [8]. In the cases with intra-articular varus deformity, the outcomes of HTO were not always satisfactory. Previous report has suggested that OWHTO cannot fully correct varus deformity in cases with JLCA greater than 6° [7, 9]. A higher risk of surgical failure led by severe malalignment and advanced knee OA has also been reported [4, 10]. Thus, HTO is considered preferable to mild to moderate medial knee OA with high joint stability remained.

Different from HTO, tibial condylar valgus osteotomy (TCVO) is able to make adaptive adjustment on the joint surface and restore the intra-articular stability. However, the uncertainty still lies in its indications. In previous reports, higher rate of undercorrected knees after OWHTO was reported in patients with pre-operative femorotibial angle (FTA) over 185° [10, 11]. To the best of our knowledge, no study has compared the outcomes of TCVO and OWHTO performed for cases with pre-operative FTA over 185°. This study aimed to present a comprehensive comparison between two types of osteotomy in terms of radiological assessment and clinical evaluations, and it also shed new insight on the indication criteria for TCVO.

## Patients and methods

### Patients selection

We retrospectively evaluated the patients who received osteotomy in our institute from September 2015 to October 2018. Anteroposterior long-leg weight-bearing radiographs were evaluated pre-operatively for calculating the correction angle. Inclusion criteria for osteotomy were symptomatic medial unicompartmental knee OA with varus malalignment, flexion contracture < 10°, range of flexion > 90°, and near-normal lateral femorotibial compartment. Exclusion criteria included lateral OA, inflammatory or rheumatoid arthritis, and smoking status. A total of 63 OA knees from 46 cases, with complete medical records and a minimum follow-up of 26 months, were included. Background characteristics are shown in Table 1. Thirty-four OA knees from 22 cases with lateral joint dilation (JLCA over 4°) and depression of the medial tibial plateau (MTPD less than − 4°) underwent TCVO surgery. OWHTO was performed on the rest cases (29 OA knees). Kellgren–Lawrence (K/L) grading system was used to evaluate the grade of osteoarthritis on the basis of measurements on a standing posteroanterior radiograph [12]. According to the K/L grading system, 15 knees (24%) were identified as grade II, 29 (46%) as grade III, and 19 (30%) as grade IV. For comparing the outcomes of these surgical procedures during severe varus deformity, 27 knees with a pre-operative FTA ≥ 185° were defined as those with severe varus (subgroup S, 15 in S_TCVO_ group and 12 in S_HTO_ group).

**Table 1:** Pre-operative characteristics

	TCVO group	HTO group	S_TCVO_ group	S_HTO_ group
Cases (male/female)	22(10/12)	24(12/12)	9(3/6)	10(4/6)
Knees (male/female)	34(16/18)	29(14/15)	15(6/9)	12(5/7)
Age (years)	56 (40–74)	59 (43–75)	57 (45–68)	61 (51–71)
BMI (kg/m^2^)	26.0 (21.1–28.5)	25.4 (23.5–27.6)	27.1 (22.2–28.2)	26.1 (24.2–27.3)
K-L grade(II,III,IV)	3, 19, 12	12, 10, 7 ^a^	0, 6, 9	1, 4, 7
Follow-up months	35 (27–42)	33 (27-65)	34 (29–41)	34 (28–39)

*BMI* Body mass index, *S*_*TCVO*_ *group/S*_*HTO*_ *group* the knee with pre-operative femorotibial angle (FTA) ≥ 185°, *K-L* Kellgren–Lawrence

^a^*P* < 0.05 compared to that before TCVO

Approval was obtained by the research ethics committee at Shanghai Jiao Tong University Affiliated Sixth People’s Hospital. All patients were informed consent to participate and approved the publication of their data.

### Surgical intervention

**Fig. 1:**
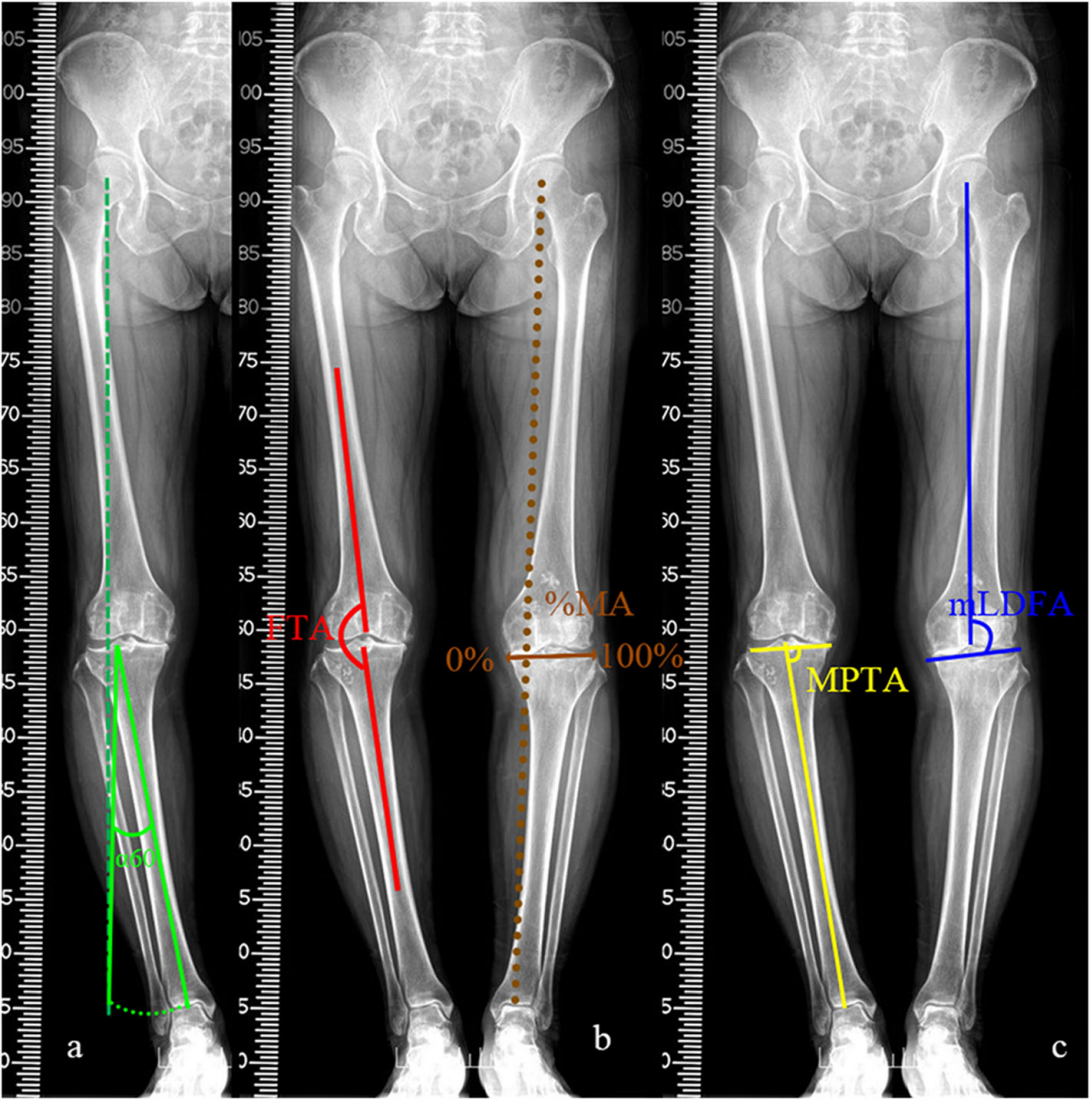
The ideal correction angle α60 was defined as the angle formed between the first line drawn from the lateral tip of intercondylar eminence to the ankle center, and the second line from the lateral tip of intercondylar eminence to the line passing through the 60% point of %MA, with the length the same as the first line (**a**). Measurement of lower limb alignment. FTA was defined as the lateral angle between the femoral anatomical shaft axis and tibial anatomical shaft axis. %MA was defined as the point where the mechanical axis intersects the tibial plateau, converted to a percentage from medial edge (0%) to lateral edge (100%) (**b**). MPTA was defined as the angle between the articular surface of the proximal tibia and the tibial mechanical axis. mLDFA was defined as the angle formed between the articular surface of the distal femur and the mechanical axis of the femur (**c**)

To obtain satisfactory lower limb alignment, pre-operative planning aims to achieve the weight-bearing line that passes through the 60% point of %MA. As shown in Fig. 1a, the ideal correction angle α60 for TCVO was defined as the angle formed between the first line drawn from the lateral tip of intercondylar eminence to the ankle center, and the second line from the lateral tip of intercondylar eminence to the weight-bearing line passing through the 60% point of %MA. The length of the second line was the same as the first line. %MA was defined as the ratio of the distance from the point of the weight-bearing line at the knee joint to the medial edge of the tibial plateau and the width of the tibial plateau. The medial margin of the tibial plateau was 0% and the lateral margin was 100% (Fig. 1b). All the operations were performed by a single senior surgeon who specialized in knee osteotomies. During the operation, the patient was placed in the supine position and a tourniquet was applied. A rod connecting the hip center to the ankle center was used to evaluate limb alignment and reassess the correction angle which ensures a post-operative %MA of 60%.

### OWHTO

The OWHTO procedure was carried out as described by Pipino et al. [5]. Briefly, a 6- to 8-cm longitudinal incision was made anteromedially along the proximal tibia. For decompression of the medial compartment, the superficial fibers of the medial collateral ligament (MCL) were dissected off the posteromedial cortex and a distal release was performed. A blunt Hohmann retractor was inserted around the posterior tibial cortex to protect the neurovascular structures. After identifying the tibial tuberosity, a guide wire was obliquely inserted from a point 4 cm below the medial joint line and passed 1 cm below the lateral articular margin of the tibia towards the fibular head, aiming at indicating the starting point of the ascending cut of the biplanar osteotomy, which prevents the osteotomy extending into tibial tuberosity. The second guide wire was placed posterior to the first guide wire, with the position parallel to each other. The horizontal osteotomy was performed at the posterior two-thirds of the tibia under fluoroscopic guidance. During opening the osteotomy gap, the lateral cortex with the distance up to 1 cm was left intact, which served as a hinge point. The subsequent ascending cut of the biplanar osteotomy started in the anterior one-third of the proximal tibia at an angle of 100° to the horizontal osteotomy plane. The bone at the osteotomy site was gradually opened with chisels and spreaders until the desired degree of correction had been achieved, as assessed by intraoperative fluoroscopy and checked further with a rigid rod connecting the centers of the hip and ankle. It should be mentioned that the anterior osteotomy gap in front of the spreader was approximately two-thirds of the posterior gap, for maintaining the posterior tibial slope. Subsequently, a TomoFix™ plate with interlocking screws was used for internal fixation of the osteotomy site. Before the final fixation, lower limb axis was further confirmed by intra-operative fluoroscopy. The osteotomized gap was grafted with artificial bone substitute comprising hydroxyapatite with beta-tricalcium phosphate (β-TCP). Finally, surgical incisions were closed layer wise.

### TCVO

**Fig. 2:**
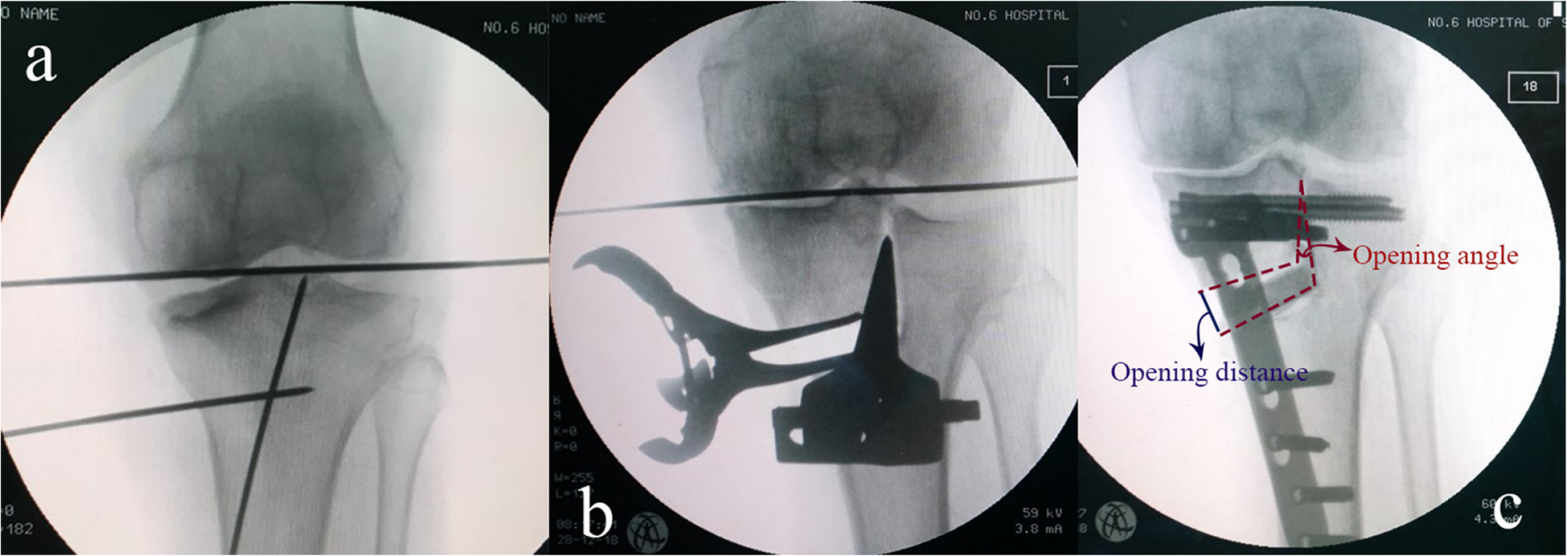
Fluoroscopic intra-operative views of a left knee underwent TCVO. **a** Two guide wires were parallelly inserted at a same horizontal level 3 cm below the joint line, with one positioned at anterior 1/3 and the other at posterior 1/3 of the tibial shaft. Another guide wire was inserted from anteroinferior site of the tibial tubercle and passed towards the tip of the lateral intercondylar eminence. **b** A spreader was applied to provide valgus force for gradually raising the lateral tibial condyle. **c** Measurement of opening angle and distance during osteotomy surgery. The distance between the intersections of the osteotomy lines with the medial cortex was defined as opening distance. The angle formed between the margins of osteotomy gap was defined as the opening angle. Application of two prophylactic hollow screws across the intercondylar eminence before fixing the locking plate is essential for protection against fracture under large correction angle

During this surgical procedure, a 5-cm skin incision was made horizontally from the medial aspect of the tibial tuberosity towards the medial border of the patellar tendon and then turned distomedially. The superficial layer of the medial collateral ligament (MCL) and pes anserinus were released, which was especially important for the patients with contracture of medial collateral ligament. Then, two guide wires were parallelly inserted at the same horizontal level 3 cm below the joint line, with one positioned at anterior 1/3 and the other at posterior 1/3 of the tibial shaft. Another guide wire was inserted from the anteroinferior site of the tibial tubercle and passed towards the center of the intercondylar eminence (Fig. 2). The “L” shaped osteotomy was performed horizontally from the medial tibial tuberosity and was then moved up vertically towards the lateral tip of intercondylar eminence with a chisel under fluoroscopic guidance. For protection of the meniscus during the surgery, the articular surface at the hinge point was maintained intact from incision, or at least, left the outer layer intact. Total incision of the articular surface at the hinge point should be avoided. Subsequently, a spreader was applied to carefully provide valgus force for gradually opening the gap at the osteotomy site to the desired correction degree planned pre-operatively. The spreader was put at the posterior cortical bone to avoid the posterior tilt of tibia slope. Intraoperative fluoroscopy was used to confirm the completion of the osteotomy. The anteromedial aspect of the tibia was fixed by a T-shaped locking plate or a Tomofix^TM^ locking plate and stabilized with locking screws. It should be noted that application of two prophylactic hollow screws across the intercondylar eminence before fixing the locking plate is essential for protection against fracture under a large correction angle (Fig. 2c). Artificial bone substitute comprising hydroxyapatite with β-TCP was used to fill the osteotomy gap for the prevention of bleeding. Surgical incisions were closed layer wise finally.

### Post-operative recovery

Rehabilitation started from the first day after OWHTO or TCVO, including isometric quadriceps exercises and range-of-motion exercises. For patients with OWHTO, partial weight-bearing with crutches was allowed at 1 week post-operatively depending on pain and wound healing, and full weight-bearing was encouraged at 4 weeks post-operatively. For patients receiving TCVO, full weight-bearing was recommended 1 week after surgery.

### Data collection and evaluation of surgical outcomes

**Fig. 3:**
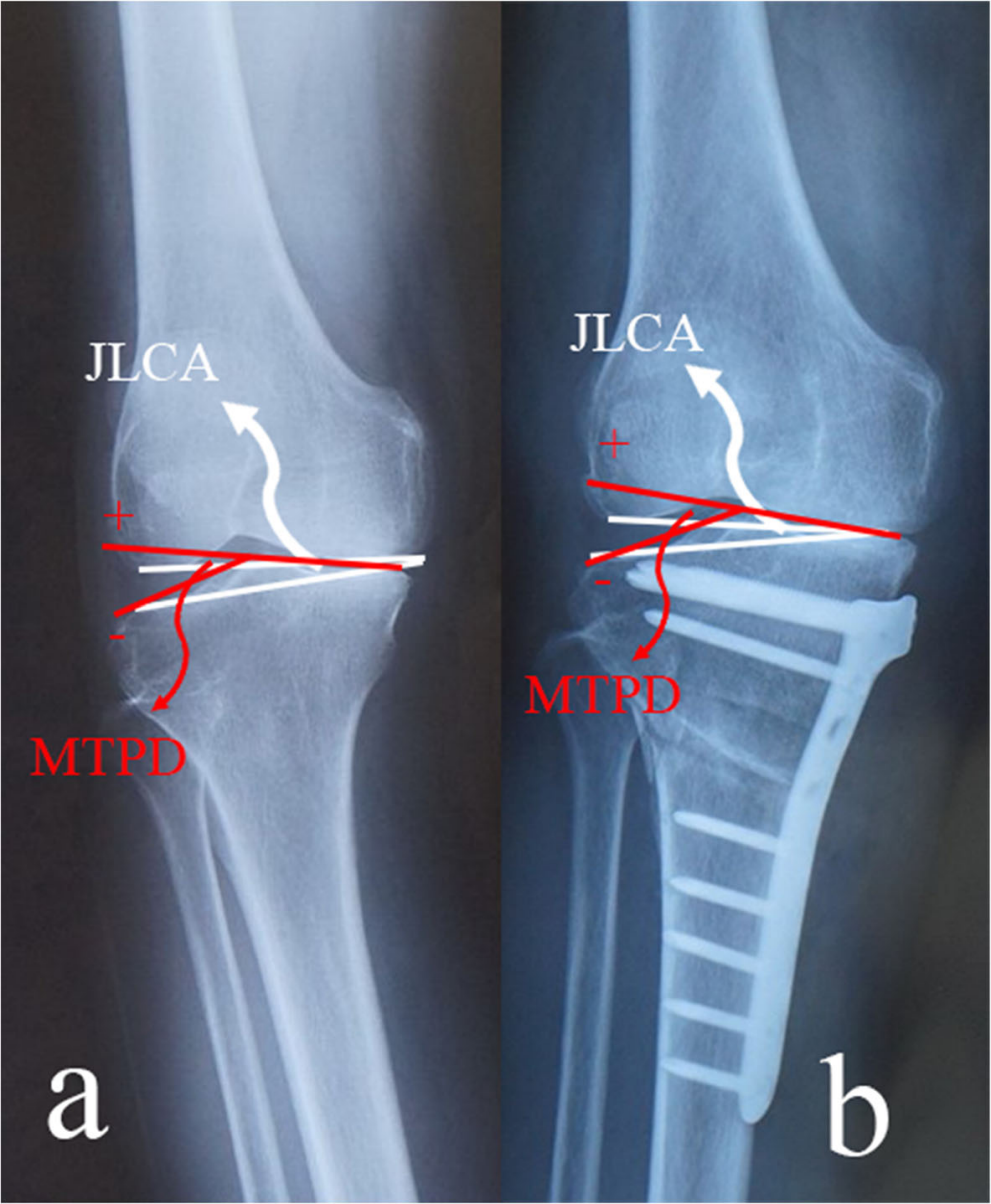
In cases of severe knee OA with inclined medial tibia plateau (decreased MTPD) and increased joint line convergence angle (JLCA) (**a**), joint instability caused by the teeter effect still exists after the surgery, no matter how OWHTO adjusts the mechanical axis laterally (**b**)

**Fig. 4:**
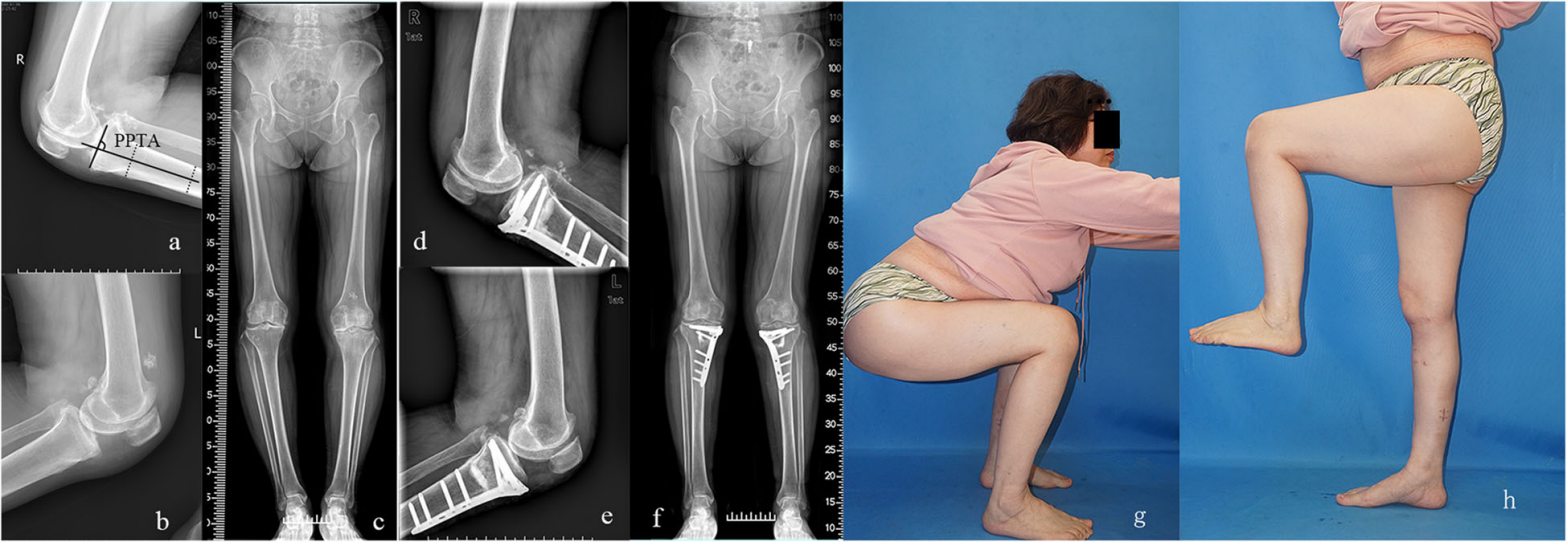
A 58-year-old female underwent tibial condylar valgus osteotomy (TCVO). Pre-operative lateral radiographs of the right (**a**) and the left (**b**) knee joints. PPTA was defined as posterior proximal tibial angle (**a**). Pre-operative standing radiograph of the lower extremities (**c**). Post-operative lateral radiographs of the right (**d**) and the left (**e**) knee joints at 2 years after surgery. Post-operative standing radiograph of the lower extremities at 2 years after surgery (**f**). She was able to jog and squat after TCVO

Radiographs obtained before and 24 months after surgery were used for evaluation of the morphology of the distal femur and proximal tibia. As shown in Fig. 1, the lower limb alignment was measured as femorotibial angle (FTA, Fig. 1b), percentage of mechanical axis on the tibial plateau (%MA, Fig. 1b), medial proximal tibial angle (MPTA, Fig. 1c), and mechanical lateral distal femoral angle (mLDFA, Fig. 1c). Measurement of joint line convergence angle (JLCA, the angle between joint orientation lines tangential to the tibial plateau and the distal point of femoral condyle on anteroposterior radiographs) was used to assess joint congruency (Fig. 3). Medial tibial plateau depression (MTPD) was used for evaluating depression angle of medial tibial plateau (Fig. 3). The range of motion (ROM) was checked before and after surgery. Posterior proximal tibial angle (PPTA) was used to evaluate the tilt of the tibia plateau at the sagittal plane (Fig. 4a). Measurement of the open wedge size (opening distance and angle) was approached by a ruler just before bone grafting and confirmed by plotting the opening wedge on the radiograph during each surgery (Fig. 2c). Opening distance referred to the distance between the intersections of the osteotomy lines with the medial cortex during gap opening. Opening angle was defined as the angle formed between two osteotomy lines after osteotomy gap opening. Each patient’s radiographs were evaluated independently by two experienced observers twice. Observers were blinded to the initial measurements, and mean values were taken as the measured values.

### Statistical analysis

Statistical analysis was performed using SPSS Statistics version 22. The data were assessed for normality using the Shapiro–Wilk test. Differences between groups were determined by Student’s *t* tests for continuous variables with a normal distribution and by Mann–Whitney test for continuous variables that were not normally distributed. Comparisons before and after surgery were determined by paired *t* tests for continuous variables with a normal distribution and Wilcoxon tests for continuous variables that were not normally distributed. Fisher’s exact probability test were used for analyzing the frequency of undercorrected knees in each group. A value of *p* < 0.05 was considered statistically significant.

## Results

The interobserver and intraobserver reliabilities for radiographic parameters and clinical scores were all satisfactory. No cases of major and minor complications were observed, except for one skin irritation in OWHTO group. Radiological features of joint congruency and limb alignment between TCVO and OWHTO were summarized in Table 3.

In terms of joint congruency, pre-operative JLCA was higher and pre-operative MTPD was significantly lower in the TCVO group than in the OWHTO group (JLCA 6.4 ± 1.7° and 2.8 ± 0.9°; MTPD − 8.0 ± 2.9° and − 1.3 ± 1.6°, respectively, *p* < 0.05). JLCA was slightly restored and MTPD was relatively the same before and after OWHTO. In TCVO group, JLCA decreased to 1.2 ± 0.9° and MTPD increased to 5.9 ± 2.5° (*p* < 0.05). PPTA decreased after OWHTO (from 83.9 ± 1.9° to 81.2 ± 2.4°, *p* < 0.05). However, no significant difference in PPTA was found before and after TCVO. In terms of limb alignment, pre-operative %MA was significantly lower in the TCVO group (5.4% ± 8.4%) than in the OWHTO group (15.9% ± 12.6%), and its value increased in both groups (*p* < 0.05).

The mean pre- and post-operative range of motion were not statistically different in the two groups, except for the difference between pre- and post-operative flexion angle in TCVO group (123.6 ± 12.6° and 134.9 ± 10.0°, *p* < 0.05), and for the difference in pre-operative flexion angle between OWHTO and TCVO group (133.9 ± 9.8° and 123.6 ± 12.6°, *p* < 0.05) (Table 2 and Fig. 4).

**Table 2:** Pre- and post-operative clinical scores and range of movement

	HTO	TCVO	S_HTO_	S_TCVO_
VAS score (points)				
Pre-operative	5.9 ± 1.3	6.0 ± 1.6	6.3 ± 1.4	6.9 ± 1.4
6-month post-operative	3.9 ± 1.4 ^a^	3.4 ± 1.2 ^a^	4.3 ± 1.1 ^a^	4.0 ± 1.1 ^a^
12-month post-operative	2.4 ± 1.4 ^a,b^	2.1 ± 1.3 ^a,b^	3.3 ± 1.2 ^a,b,c^	1.9 ± 1.0 ^a,b^
24-month post-operative	2.6 ± 1.5 ^a,b^	2.1 ± 1.2 ^a,b^	3.5 ± 1.4 ^a,e^	2.2 ± 0.9 ^a,b^
HSS score (points)				
Pre-operative	70.3 ± 6.2	65.9 ± 8.3	67.5 ± 4.8	63.2 ± 8.1
6-month post-operative	77.3 ± 4.9 ^a^	76.8 ± 6.5 ^a^	73.8 ± 2.2 ^a^	76.2 ± 8.6 ^a^
12-month post-operative	82.7 ± 6.0 ^a,b^	86.2 ± 7.2 ^a,b^	78.6 ± 2.8 ^a,b,c^	85.9 ± 7.9 ^a,b^
24-month post-operative	81.4 ± 6.6 ^a,b^	87.3 ± 7.3 ^a,b^	75.3 ± 3.3 ^a,e^	86.7 ± 7.4 ^a,b^
Range of movement				
Pre-operative				
Extension angle (°)	− 3.7 ± 3.2	− 3.6 ± 1.8	− 3.4 ± 3.1	− 3.4 ± 2.1
Flexion angle (°)	133.9 ± 9.8 ^d^	123.6 ± 12.6	131.9 ± 8.2 ^d^	117.7 ± 12.9
24-month post-operative				
Extension angle (°)	− 3.0 ± 1.6	—3.5±2.0	− 3.0 ± 2.0	− 3.5 ± 2.5
Flexion angle (°)	134.9 ± 8.1	134.9±10.0 ^a^	132.9 ± 8.5	135.3 ± 10.3 ^a^

*VAS* Visual analog scale, *HSS* Hospital for Special Surgery, *S*_*TCVO*_ *group / S*_*HTO*_ *group* the knee with pre-operative femorotibial angle (FTA) ≥ 185°.

^a^*P* < 0.05 compared to that pre-operatively

^b^*P* < 0.05 compared to that 6-month post-operatively

^c^*P* < 0.05 compared to that 12 months after TCVO

^d^*P* < 0.05 compared to that before TCVO

^e^*P* < 0.05 compared to that 24 months after TCVO

Pre-operative FTA was slightly higher in the TCVO group than that in the OWHTO group (185.0 ± 3.8° and 182.9 ± 3.9°). The same trend was also observed in their subgroups (188.5 ± 2.9° and 186.7 ± 1.2°). As shown in Table 3, S_HTO_ and S_TCVO_ group showed inferior results in terms of pre-operative clinical scores and radiographic parameters when compared with that of the overall cases. The average post-operative FTA in the S_TCVO_ group (171.1 ± 2.9°) was lower than that in the S_HTO_ group (176.3 ± 2.0°) (*p* < 0.05). The average post-operative knee flexion was 135.3 ± 10.3° in the S_TCVO_ group and 132.9 ± 8.5° in the S_HTO_ group (*p* < 0.05). Clinical assessment by the VAS and HSS scores revealed improvements of these patients at 12 and 24 months after TCVO, which were superior to those after OWHTO. A higher rate of undercorrected knees (post-operative FTA over 175°) was observed in the S_HTO_ than that in the S_TCVO_ groups (58.3% in S_HTO_ group and 13.3% in S_TCVO_ group, *p* < 0.05).

**Table 3:** Pre- and post-operative radiographic features

	HTO	TCVO	S_HTO_	S_TCVO_
%MA				
Pre-operative	15.9 ± 12.6^a^	5.4 ± 8.4	6.9 ± 6.1^a^	− 1.6 ± 5.1
24-month post-operative	59.7 ± 10.1^b^	61.2 ± 7.1^a^	51.4 ± 8.5^b,c^	61.3 ± 7.6^a^
FTA (°)				
Pre-operative	182.9 ± 3.9	185.0 ± 3.8	186.7 ± 1.2	188.5 ± 2.9
24-month post-operative	172.9 ± 3.4^b^	171.3 ± 2.5^a^	176.3 ± 2.0^b,c^	171.1 ± 2.9^a^
MPTA (°)				
Pre-operative	82.4 ± 2.9	82.4 ± 2.8	80.2 ± 1.6	80.9 ± 2.9
24-month post-operative	90.3 ± 2.4 ^b^	91.7 ± 2.1^a^	88.2 ± 1.6^b,c^	92.8 ± 2.0^a^
mLDFA (°)				
Pre-operative	88.6 ± 1.2	88.9 ± 1.3	88.7 ± 1.2	89.2 ± 1.2
24-month post-operative	88.1 ± 1.0	88.6 ± 1.6	88.4 ± 0.9	89.5 ± 1.3
JLCA (°)				
Pre-operative	2.8 ± 0.9^a^	6.4 ± 1.7	3.6 ± 0.3^a^	7.5 ± 1.6
24-month post-operative	2.2 ± 1.3^b^	1.2 ± 0.9^a^	3.4 ± 1.1 ^c^	1.4 ± 1.1^a^
MTPD (°)				
Pre-operative	− 1.3±1.6 ^a^	− 8.0 ± 2.9	− 2.4 ± 1.1^a^	− 9.9 ± 2.6
24-month post-operative	− 1.0±1.8 ^c^	5.9 ± 2.5^a^	− 2.3 ± 1.4^c^	5.9 ± 3.2^a^
PPTA (°)				
Pre-operative	83.9 ± 1.9	82.4 ± 2.4		
24-month post-operative	81.2 ± 2.4^b^	82.0 ± 3.2		

*%MA* percentile of the lower limb mechanical axis on the tibial plateau, *FTA* Femorotibial angle, *MPTA* Medial proximal tibial angle, *mLDFA* Mechanical lateral distal femoral angle, *JLCA* Joint line convergence angle, *MTPD* Medial tibial plateau depression angle, *PPTA* Posterior proximal tibial angle

^a^*P* < 0.05 compared to that before TCVO

^b^*P* < 0.05 compared to that before HTO

^c^*P* < 0.05 compared to that 24 months after TCVO

Bony fragment in TCVO group underwent larger distance of opening (14.0 ± 4.5 mm) during TCVO, while in OWHTO group such distance was relatively shorter (10.1 ± 2.9 mm, *p* < 0.01) (Table 4). Regarding the difference in the change of MPTA in each case, opening distance per △MPTA (mm) was also calculated. Compared with the value in OWHTO group, such value was significantly larger in TCVO group (*p* < 0.01). The comparison of opening angle and opening angle per △MPTA also followed the same trend.

**Table 4:** Opening distance and angle of the osteotomy gap during two operations

	HTO	TCVO	*p* value
△MPTA (°)	7.9 ± 2.3	9.3 ± 3.2	< 0.05
Opening distance (mm)	10.1 ± 2.9	14.0 ± 4.5	< 0.01
Opening distance per △MPTA (mm)	73.6 ± 4.1	86.4 ± 2.7	< 0.01
Opening angle (°)	9.3 ± 2.5	19.1 ± 5.8	< 0.01
Opening angle per △MPTA	1.2 ± 0.1	2.1 ± 0.2	< 0.01

*△MPTA* the change of MPTA after each surgery (△MPTA = post-operative MPTA−pre-operative MPTA), *opening distance* the distance between the intersections of the osteotomy lines with the medial cortex during gap opening, *opening distance per △MPTA* = opening distance × 180÷(△MPTA × π), *opening angle* the angle formed between two osteotomy lines after osteotomy gap opening, *opening angle per △MPTA* = opening angle ÷ △MPTA

## Discussion

With the progress of knee OA, the medial meniscus and articular cartilage will tear or even be absent, which causes inclination of the medial tibial plateau and narrowed medial joint gap (decreased MTPD and increased JLCA), and may eventually lead to joint subluxation. The outcome of OWHTO procedure has been reported to be far beyond satisfaction in the cases with teeter effect and lateral thrust phenomenon [7, 8]. As shown in Fig. 3, although OWHTO shifted the mechanical axis (%MA) laterally to the same extent as TCVO, the joint facet could only relate with each other to a limited extent and intra-articular incongruency caused by the teeter effect still exists after the surgery. However, after TCVO, the femoral condyle was wedged from both sides as the shape of the tibial plateau became more concave, which restored the inclined medial joint surface and readjusted the widened lateral joint space (Fig. 4). Such findings indicate that TCVO can improve the joint congruency of the affected knee and works better in intra articular deformity with increased depression of medial tibial plateau (MTPD less than − 4°), and widened lateral joint (JLCA over 4°).

The advanced OA usually results in the laxity of the medial collateral ligament (MCL) and increased tension of lateral collateral ligament (LCL), which aggravates joint stability. During TCVO, we discovered that elevation of the medial tibia plateau could help recover the tension of MCL and subsequently loosen the LCL as the lower limb alignment was restored. Furthermore, a previous study also reported increased tension in the cruciate ligaments which contributed to increased joint stability [13]. Thus, TCVO does not require additional ligament reconstruction to improve joint stability and congruency.

Previous studies have reported a significant decrease in PPTA after OWHTO [14, 15]. However, in our study, a relatively mild decrease of PPTA was achieved in OWHTO group. In fact, we managed to modify the posterior tilt angle of tibia plateau through the biplane osteotomy during OWHTO. The key point lies in the position of the spreader during gap opening. The tibial plateau tends to tilt posteriorly if the spreader is positioned relatively anteriorly. Thus, during OWHTO the anterior gap of the osteotomy in front of the spreader was set approximately two-thirds the posterior gap, which maintained the posterior tibial slope. On the other hand, PPTA before and after TCVO remained the same as TCVO is a single plane osteotomy, which indicates that TCVO cannot deal with joint instability or abnormal knee kinematics in the anteroposterior direction. For this reason, it is difficult to improve flexion contractures using TCVO and pre-operative flexion contracture of over 10° maybe a contraindication for TCVO.

The shape of tibia plateau (“pagoda-type”, determined by JLCA and MTPD) and advanced K/L grade were the indication criteria recommended by several reports and verified in our study [13, 16]. However, to the best of our best knowledge, no study has taken the alignment of lower limb and the accurate value of the certain parameters into consideration. We noticed that some studies recommended pre-operative FTA less than 185° as a criterion for OWHTO [10, 11]. In our study, we compared the radiological and clinical results of TCVO and OWHTO applied in patients with severe varus deformity (pre-operative FTA ≥ 185°) and who were reluctant to receive arthroplasty. A significantly better improvement was observed in these patients receiving TCVO. It is worth noting that the proportion of undercorrected knees were higher after OWHTO. Varus deformity of the distal femur (increased mLDFA), varus deformity of the proximal tibia (decreased MPTA), and intra-articular varus deformity (increased JLCA) are the three major causes of varus knee (increased FTA) in OA progression [16]. For severe OA patients with pre-operative FTA of over 185° and with normal mLDFA, there is a higher frequency of intra-articular deformity with advanced K/L grade [17, 18]. However, some of these patients did not fulfill the indication criteria of TCVO due to JLCA no more than 4° and MTPD no less than − 4°. Bito et al. demonstrated that advanced degree of tibia varus resulted in a higher frequency of correction loss following OWHTO [11]. Earlier clinical practice has also shown that joint instability associated with lateral thrust, flexion contracture of over 15°, and poor active knee ROM (< 90°), might lead to undercorrection and recurrence of varus deformity [19]. Akamatsu et al. reported that coronal subluxation and the joint space angle strongly correlated with the correction loss and change in FTA after OWHTO. Their pre-operative findings also showed the correlation between coronal subluxation and FTA, a marker of knee OA severity or progression [20]. Coronal subluxation was considered to be related to the widening of the lateral joint space and the narrowing of the medial joint space [20]. Severe soft tissue laxity could increase the inconsistency between the actual and predicted alignments due to the effects of weight-bearing conditions on alignment changes following OWHTO [21]. To restore the medial joint space, the distal fibers of the MCL are surgically released during exposure of the osteotomy site before gap opening. Opening the osteotomy during surgical procedure and healing of the released MCL will retighten the medial structures. On the other hand, tensile stress led by varus forces will cause the lateral structures loosen from stretching [11]. The imbalanced stability between medial and lateral structures, along with the remained intra-articular incongruency after OWHTO procedure, produces relatively strong resistance against the knee valgus correction [20, 22]. In TCVO, as the femoral condyle is wedged from both sides into the flat-type tibial plateau, the joint space is restored and the balanced collateral structures help to stabilize and maintain the correction angle after surgery.

In satisfied post-operative alignment, the forces pass through the point lateral to the knee joint center and create an adduction moment at the lateral compartment, which opens medial space and decreases JLCA. However, for OWHTO cases with severe genu varum, the resistance against the valgus correction will cause the forces pass through the relatively medial point of the knee joint, with the adduction moment insufficient to open the medial joint space. Based on these findings and the results from other studies, the present study proposed the possibility of pre-operatively predicting whether correction loss will occur by evaluating FTA. Patients with severe varus who are physically active and reluctant to undergo TKA or UKA should be informed that OWHTO is improper for knees with severe varus. In principle, TCVO is preferable for medial-compartment OA with intra articular deformity and a pre-operative FTA ≥ 185°.

No major complications occurred in our study. Bone grafts or substitutes were applied for preventing local blood loss and facilitating bone healing, especially in cases with large gaps, poor bone quality, or obesity [23]. In our study, tricalcium phosphate (TCP) was selected as synthetic bone substitutes in both types of surgeries as it has better osteo-conductivity and absorbability than hydroxyapatite substitute and can avoid the risk of immune response or disease transmission brought about by allografts [24]. Hinge fracture is a critical factor in correction loss, instability, and delayed union [25]. In TCVO, since the lateral tibial condyle is intact, risk of hinge fracture is lower, which enables early weight-bearing after the surgery. However, under a large correction angle during TCVO, we recommend that two additional prophylactic hollow screws should be inserted across the intercondylar eminence before fixing the locking plate, which is essential for protection against avulsion of the intercondylar eminence (Fig. 2c). To protect the meniscus from iatrogenic injury, we considered the hinge point should be positioned at three places, that are the medial beak, center, and lateral beak of intercondylar eminence. The “L”-shaped osteotomy should be performed horizontally from the medial tibial tuberosity first, and then moved up towards the hinge point. Due to the flexibility of the meniscus tissue, it could be remodeled at the hinge point during gap opening. Thus, the articular surface at the hinge point should be maintained intact from incision, or at least, left the outer layer intact. Total incision of the articular surface at the hinge point should be avoided.

The mean opening distance and opening angle were relatively lower in TCVO group than in OWHTO group. Evaluation of opening distance per △MPTA (mm) and opening angle per △MPTA further revealed that larger magnitude of gap opening for raising the bony fragment was required in TCVO than that in OWHTO, when MPTA was corrected to the same extent. In other words, to obtain the same amount of MPTA correction, OWHTO has more advantages in angle correction, while such capacity is limited for TCVO. Hinge position and pre-operative deformity are associated with the degree of gap opening angle during corrective osteotomy. Up till now, there is no reliable consensus on the appropriate selection of the hinge point for TCVO. To avoid iatrogenic injury to the articular surface on tibia plateau, three positions could be selected as the hinge point, that is the medial beak, center, and lateral beak of intercondylar eminence. As TCVO could be considered as a special type of OWHTO, with its hinge point positioned more medially than that of OWHTO. We speculate that the hinge point positioned at the lateral beak of intercondylar eminence would lead to the smallest opening angle. A simulated re-alignment process with the hinge point set at these three positions respectively may help to theoretically explain the causal relationship between hinge position and magnitude of the opening gap. Larger opening angle has been reported to be associated with greater risks of hinge fracture either intra-operatively or post-operatively [26, 27]. Mismatch between the contact area of locking plate and post-correction bony surface may also occur as the horizontal distance between the proximal and distal fragments increases with osteotomy gap opening [28]. Yoo et al. suggested that during OWHTO, bending of the locking plate at both end of the opening gap in the coronal plane is necessary to fit the bony surface geometry and provide appropriate and effective physiological stress [29]. We consider these procedures might be also necessary for the severe genu varum receiving TCVO. To predict the inconsistency between the actual and predicted alignments, Kuwashima et al. defined the expected correction angle β by TCVO as the following: β = (varus stress JLCA + valgus stress JLCA) × 1.5. According to Kuwashima’s opinion, TCVO alone could be sufficient to correct the valgus alignment if α60 (the ideal correction angle aforementioned) was smaller than β. Otherwise, undercorrection might occur when only TCVO is performed [16]. The efficacy of this method deserves further study. Combined proximal tibial osteotomy (CPTO) with additional OWHTO performed was recommended under such cases [8, 16]. Thus, an accurate pre-operative calculation is crucial for achieving the optimal outcomes and the lateral joint needs to be over-corrected in case of severe varus deformity, so that the alignment of the lower extremity may become valgus enough.

Short-term period of follow-up remains to be one of the limitations in this study. A long-term investigation is needed to determine the indications, complications, and prognostic factors of this novel surgery. Sample size is slightly small, although the sample size of S_TCVO_ and S_HTO_ groups was comparable to that calculated with reference to relevant previous studies. A larger sample size is warranted in further study to allow sustainable statements on the effectiveness and efficiency of TCVO when the outcome of OWHTO is unsatisfied. As the pathogenesis of knee OA maybe different according to ethnic, life style, and other demographic characteristics, a prospective design including paired case and control groups, applying Propensity Score Matching (PSM) if necessary, is required to obtain representative results for the general population. Opening the osteotomy gap is a three-dimensional movement during the procedure, but we only used a ruler and confirmed the value by plotting the opening wedge on the radiograph during each surgery. Three-dimensional (3D) computed tomography maybe helpful in studying the ideal movement of osteotomized bony fragment under dynamic conditions or other more complex loading scenarios. In addition, although the difference between pre- and post-operative soft tissue laxity evaluated on varus/valgus stress radiographs maybe interesting, these data were not collected in this study due to a high false-negative rate caused by poor cooperation by patients with severe pain [21].

## Conclusion

In conclusion, TCVO is a preferable option in treating advanced knee OA with intra-articular deformity. In addition, we observed that TCVO is suitable for severe varus deformity (pre-operative FTA ≥ 185°) and increased depression of medial tibial plateau (JLCA over 4° and MTPD less than − 4°). However, compared with OWHTO, TCVO has a limited capacity in correcting the varus angle both in the coronal and sagittal planes. Appropriate patient selection, precise surgical techniques, and accurate rehabilitation protocols are all required for the success of TCVO.

## Data Availability

All data generated or analyzed during this study are included in this published article.

## Abbreviations

OA: Osteoarthritis
TCVO: Tibial condylar valgus osteotomy
OWHTO: Open wedge high tibial osteotomy
VAS: Visual analog scale
HSS: Hospital for Special Surgery
FTA: Femorotibial angle
JLCA: Joint line convergence angle
ROM: Range of motion
MTPD: Medial tibial plateau depression
%MA: Percentage of mechanical axis on the tibial plateau
MPTA: Medial proximal tibial angle
mLDFA: Mechanical lateral distal femoral angle
K/L: Kellgren–Lawrence
PPTA: Posterior proximal tibial angle
MCL: Medial collateral ligament
β-TCP: Beta-tricalcium phosphate
